# Supplementary material for: In Vivo Anticancer Evaluation of 6b, a Non-Covalent Imidazo[1,2-a]quinoxaline-Based Epidermal Growth Factor Receptor Inhibitor against Human Xenograft Tumor in Nude Mice
Source: Molecules. 2022 Aug 28;27(17):5540. doi: 10.3390/molecules27175540 (PMC9457798; doi:10.3390/molecules27175540)
Supplement: Supplementary file 1 [file molecules-27-05540-s001.zip › molecules-1881679-supplementary.pdf]

Supporting Information

In vivo anticancer evaluation of 6b, a non-covalent imidazo[1,2-a]quinoxaline-based epidermal growth factor receptor inhibitor against human xenograft tumor in nude mice

Zahid Rafiq Bhat[1, †], Manvendra Kumar[2, †], Nisha Sharma[1], Umesh Prasad Yadav[3], Tashvinder Singh[3], Gaurav Joshi[2], Brahmam Pujala[4], Mohd. Raja[4], Joydeep Chatterjee[2], Kulbhushan Tikoo[1, *], Sandeep Singh[3, *], and Raj Kumar[2, *]

[1] Department of Pharmacology and Toxicology, National Institute of Pharmaceutical Education and Research, S.A.S. Nagar, Punjab-160062, India.
[2] Laboratory for Drug Design and Synthesis, Department of Pharmaceutical Sciences and Natural Products, School of Health Sciences, Central University of Punjab, Bathinda 151 401, India.
[3] Laboratory of Molecular Medicine, Department of Human Genetics and Molecular Medicine, Central University of Punjab, Bathinda 151401.
[4] Integral BioSciences Pvt. Ltd.C-64, Hosiery Complex, Phase-II, Noida-201306, UP, India.

Spectra for compound 6b

^{1}H NMR

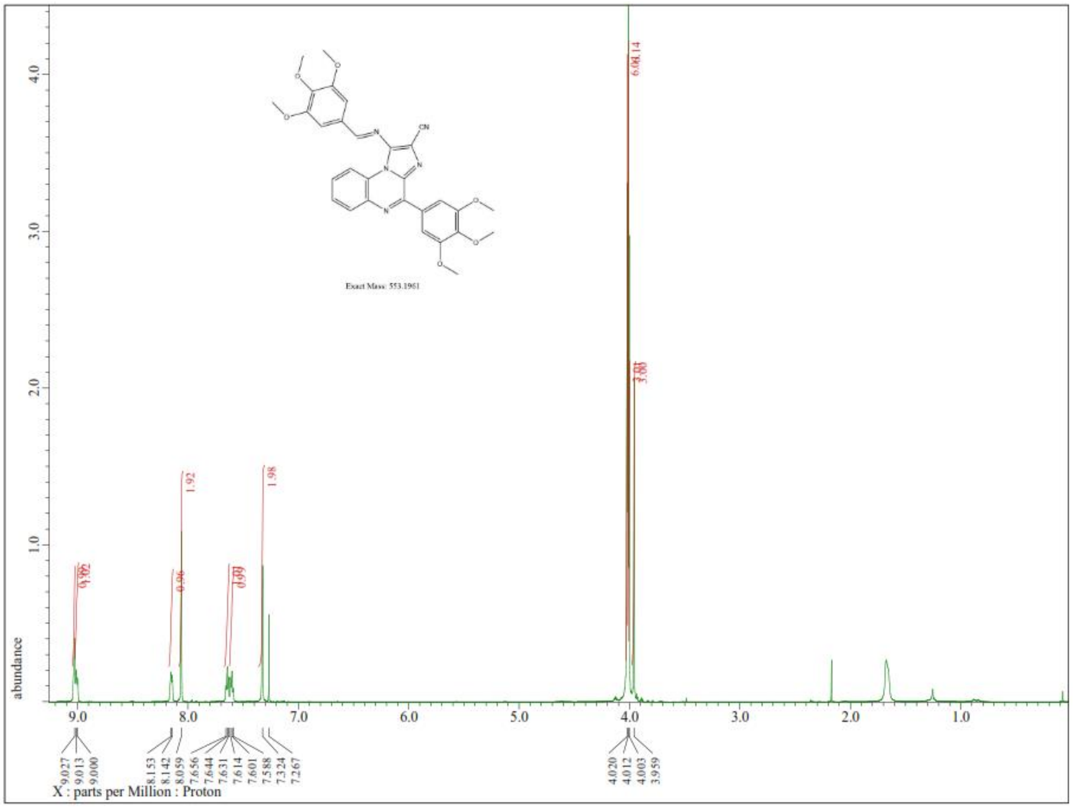

Figure S1: Proton NMR of target compound (**6b**)

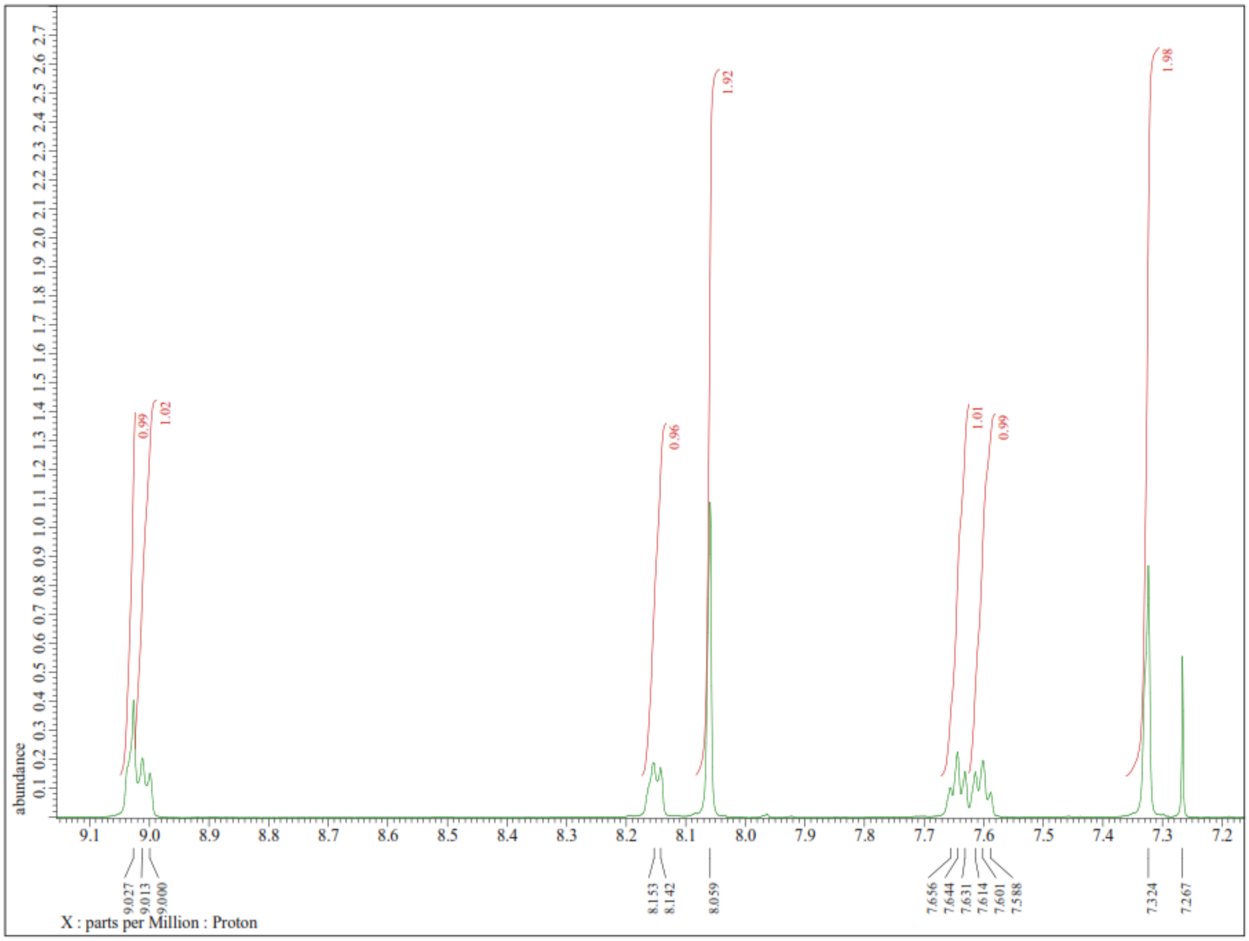

abundance
X : parts per Million : Proton

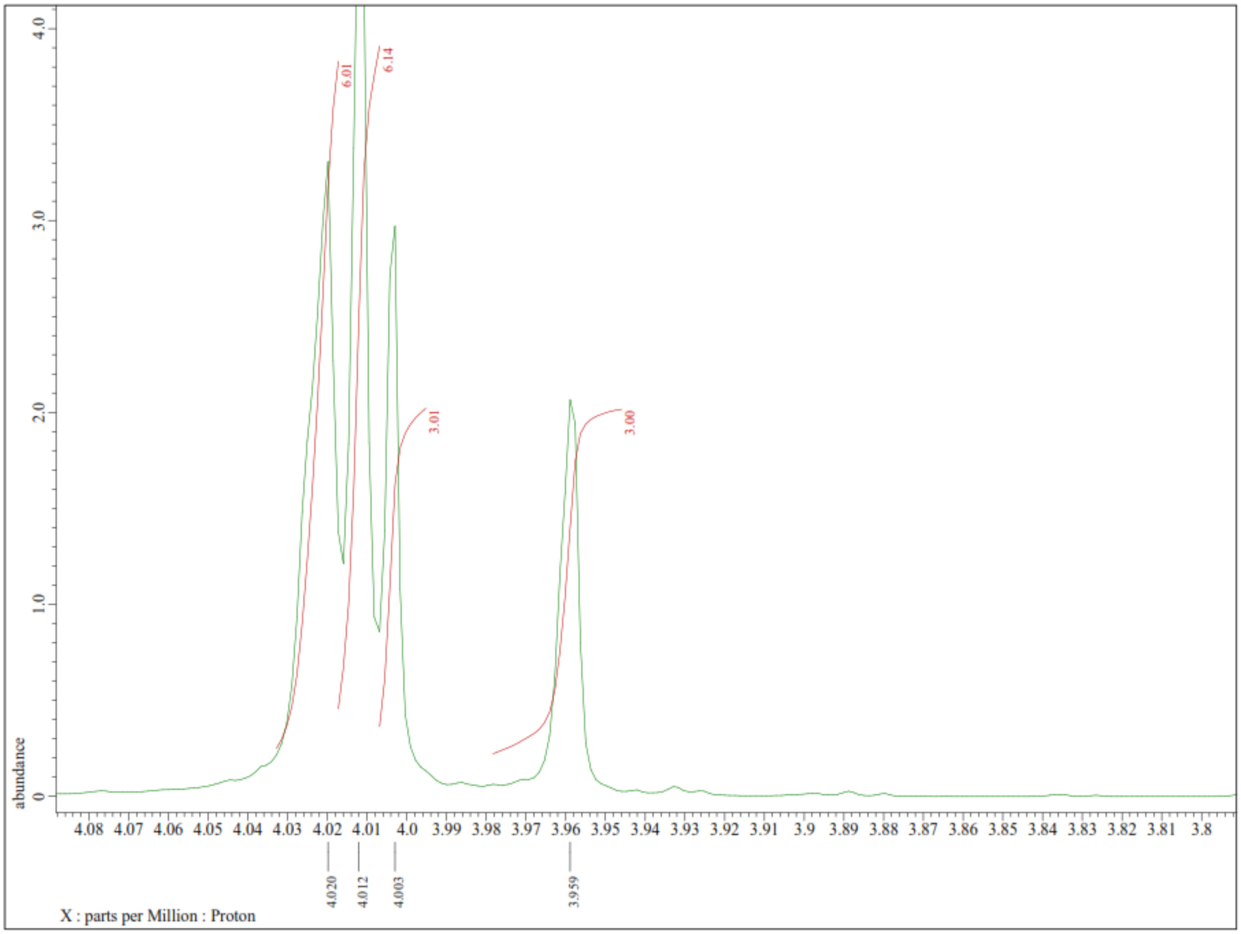

abundance
0
1.0
2.0
3.0
4.0
6.01
6.14
3.01
3.00
4.08 4.07 4.06 4.05 4.04 4.03 4.02 4.01 4.0 3.99 3.98 3.97 3.96 3.95 3.94 3.93 3.92 3.91 3.9 3.89 3.88 3.87 3.86 3.85 3.84 3.83 3.82 3.81 3.8
4.020
4.012
4.003
3.959
X : parts per Million : Proton

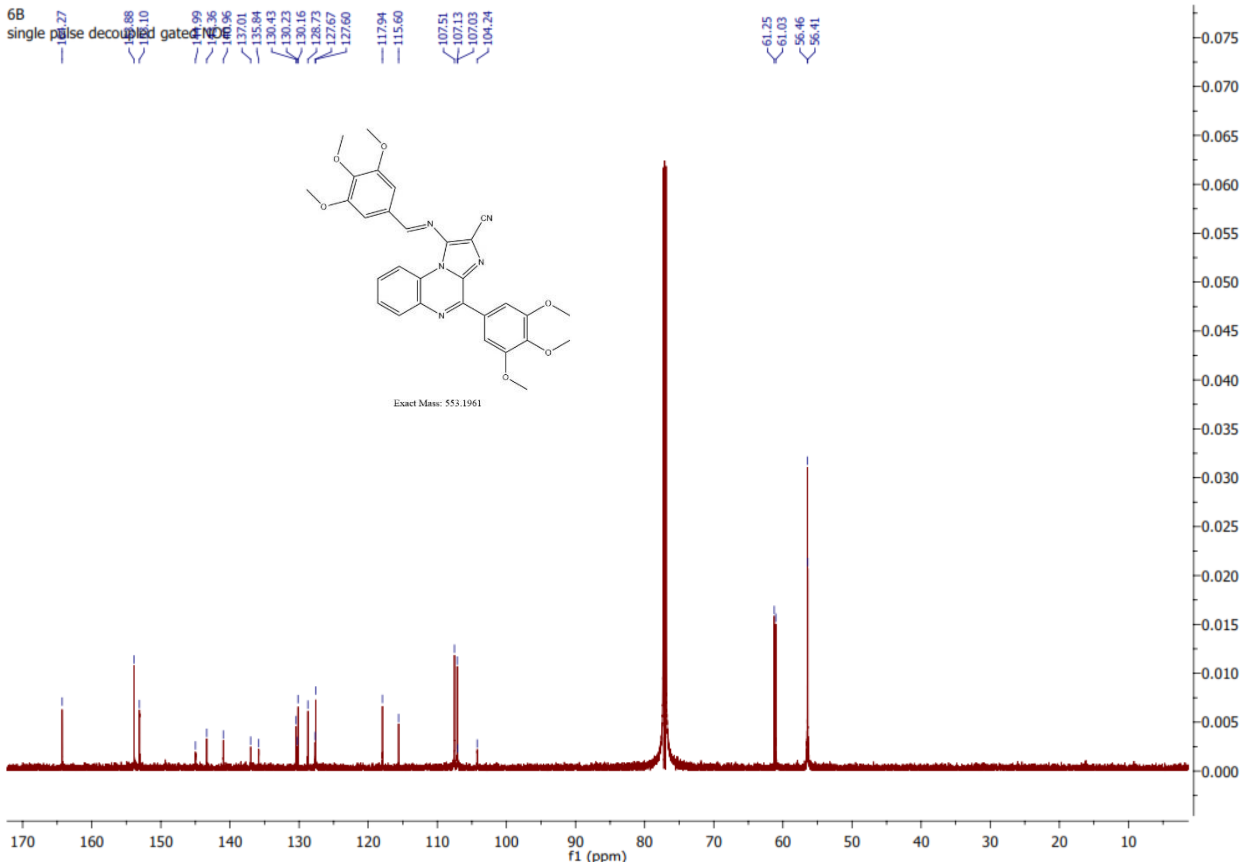

Figure S2: Carbon NMR of target compound (**6b**)

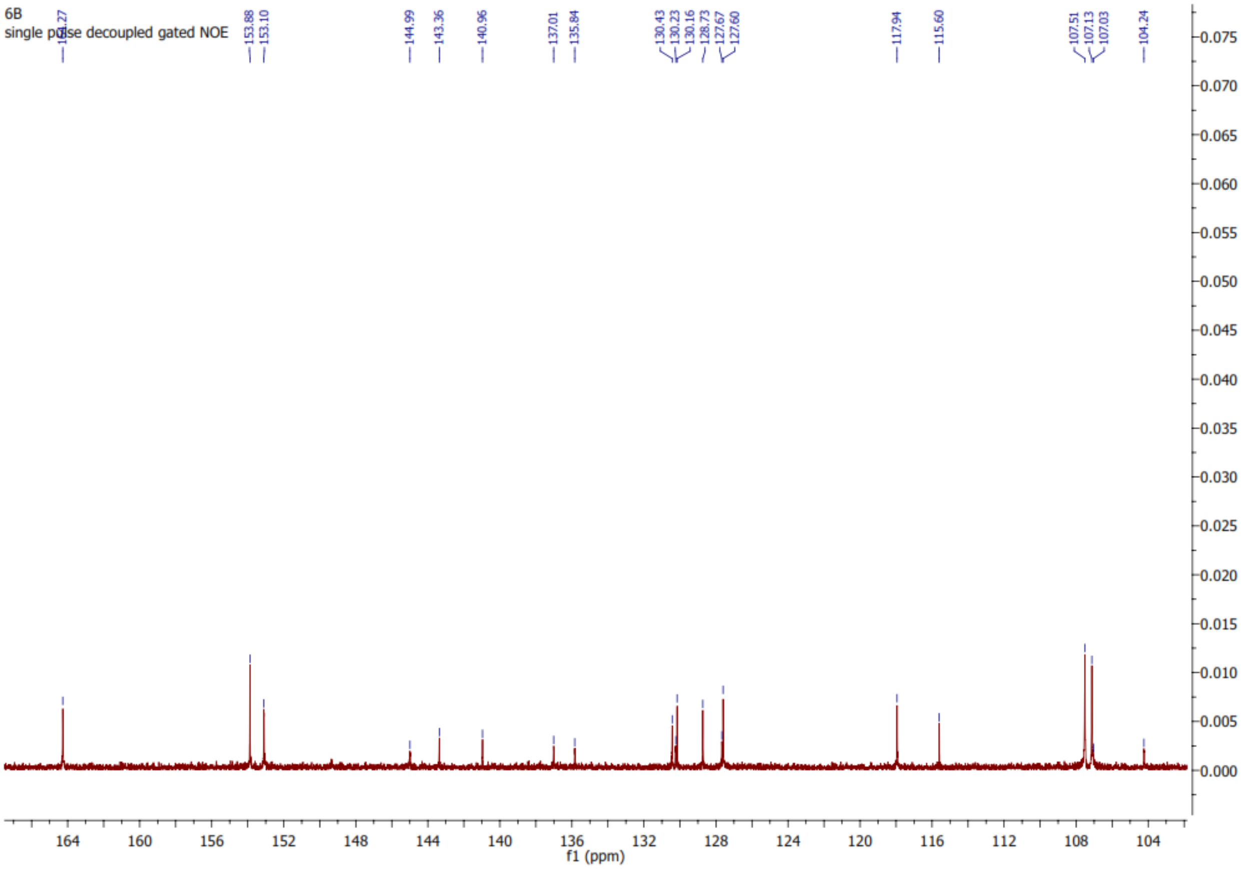

6B
single pulse decoupled gated NOE
164.27
153.88
153.10
144.99
143.36
140.96
137.01
135.84
130.43
130.23
130.16
128.73
127.67
127.60
117.94
115.60
107.51
107.13
107.03
104.24
f1 (ppm)

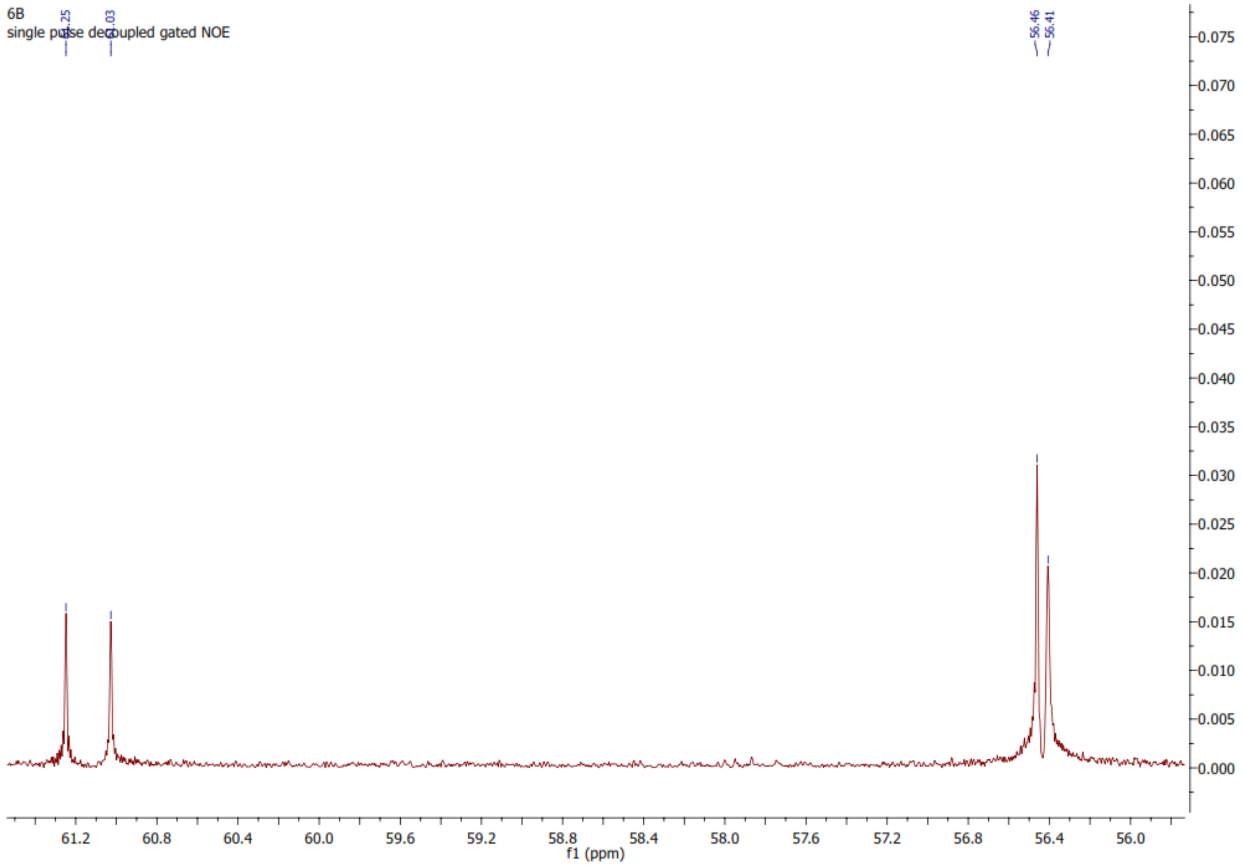
6B
single pulse decoupled gated NOE
61.25
61.03
56.46
56.41
f1 (ppm)
